# Prognostic Value of miR-21 in Various Cancers: An Updating Meta-Analysis

**DOI:** 10.1371/journal.pone.0102413

**Published:** 2014-07-14

**Authors:** Xin Zhou, Xiaping Wang, Zebo Huang, Jian Wang, Wei Zhu, Yongqian Shu, Ping Liu

**Affiliations:** 1 Department of Oncology, First Affiliated Hospital of Nanjing Medical University, Nanjing, China; 2 Cancer Center of Nanjing Medical University, Nanjing, China; 3 Key Laboratory of Human Functional Genomics of Jiangsu Province, Clinical Diabetes Centre of Jiangsu Province, Nanjing Medical University, Nanjing, China; University of Aberdeen, United Kingdom

## Abstract

**Background:**

Recently, more and more studies investigated the value of microRNA (miRNA) as a diagnostic or prognostic biomarker in various cancers. MiR-21 was found dysregulated in almost all types of cancers. While the prognostic role of miR-21 in many cancers has been studied, the results were not consistent.

**Methods:**

We performed a meta-analysis to investigate the correlation between miR-21 and survival of general cancers by calculating pooled hazard ratios (HR) and 95% confidence intervals (CI).

**Results:**

The pooled results of 63 published studies showed that elevated miR-21 was a predictor for poor survival of general carcinomas, with pooled HR of 1.91 (95%CI: 1.66–2.19) for OS, 1.42 (95% CI: 1.16–1.74) for DFS and 2.2 (95% CI: 1.64–2.96) for RFS/CSS. MiR-21 was also a prognostic biomarker in the patients who received adjuvant therapy, with pooled HR of 2.4 (95%CI: 1.18–4.9) for OS.

**Conclusions:**

Our results showed that miR-21 could act as a significant biomarker in the prognosis of various cancers. Further studies are warranted before the application of the useful biomarker in the clinical.

## Introduction

Due to the aging and growth of population as well as an increasing adoption of cancer-related lifestyle such as smoking and “westernized” diets, cancer has been a major public health problem all around the world [1]. Almost one in four deaths in the United States is related with cancer in 2012 [2]. Lack of efficiently diagnostic and prognostic biomarkers is responsible for the high mortality rates caused by cancer [3].

MicroRNAs (miRNAs), approximately 22 nucleotides in length, are a class of highly conserved RNAs that negatively regulate gene expression at post-transcriptional level by base pairing with the 3′-untranslated region of target mRNAs, resulting in either mRNA degradation or translational inhibition [4], [5]. Many studies have demonstrated that miRNAs play important roles in various biological processes, such as cellular development, differentiation, proliferation, cell death, angiogenesis and metabolism [6]–[9]. The success of utilizing miRNAs as diagnostic or prognostic markers from expression profiling has been reported in many studies.

MiR-21 was one of the most frequently studied cancer-related miRNAs and dysregulated in most cancers by acting as oncogene [10]–[14]. Up-regulated miR-21 could increase tumor growth, metastasis and invasion and reduce sensitivity to chemotherapy by its various targets [15]–[18]. Cancer patients with higher expression of miR-21 always had a worse prognostic outcome. But some studies represented inconsistent or even opposite results, such as the study of Valladares-Ayerbes et al. [19]. So we performed this meta-analysis to reveal the prognostic value of miR-21 in various cancers.

## Material and Methods

### Publication search and inclusion criteria

Medical subheading (Mesh) terms relating to miR-21 (e.g. “miR-21” or “microRNA-21”) in combination with words related to cancer (e.g. “cancer”, “tumor”, “carcinoma” or “neoplasm”) and terms to prognosis (e.g. “prognosis”, “survival”, “outcome” or “prognostic”) were searched on PubMed, EMBASE and WEB of science to retrieve eligible studies till December, 2013 .

We also carefully examined the references of articles and reviews to explore potentially additional studies. Studies were eligible if they met the following criteria: (a) studied patients with any type of cancers; (b) expression of miR-21 was measured; (c) the association between expression of miR-21 and clinical outcome was investigated; (d) full text articles in English. Studies were excluded based on the following criteria: (a) reviews, letters or laboratory studies; (b) studies had overlapping or duplicate data; (c) absence of key information for further analysis [20].

### Data extraction

Data were evaluated and extracted independently from the eligible studies by two investigators (Zhou and Wang) under the guidelines of a critical review checklist of the Dutch Cochrane Centre proposed by Meta-analysis of Observational Studies in Epidemiology (MOOSE) [21]. The following items were recorded: first author's name, year of publication, country or area of origin, ethnicity, cancer type, sample type, TNM stage, method, total number of patients, cut-off value, follow ups and HRs of miR-21 for overall survival (OS), disease-free survival (DFS), recurrence-free survival (RFS) or cancer specific survival (CSS) with their 95% confidence intervals (CIs) and P value. If not available, data were extracted by the method of Tierney et al. [20]. When discrepancies existed between the two investigators, another investigator (Huang) was invited to discuss until a consensus was reached.

### Statistical analysis

All the HRs with their 95% CIs were used to calculate pooled HRs. Cochran's Q test and Higgins I-squared statistic were used to check the heterogeneity of pooled results. A P<0.10 for Q-test suggested significant heterogeneity among studies, and the random-effects model (DerSimonian-Laird method) was applied to calculate the pooled HRs [22]. Otherwise, the fixed-effects model (Mantel-Haenszel method) was used [23]. Begg's funnel plot and the Egger's linear regression test were conducted to evaluate publication bias of literatures and a p<0.05 was considered significant [24]. Trim and fill method was applied to assess potential asymmetry in the funnel plot. Statistical analyses were performed in STATA software version 12.0 (STATA Corporation, College Station, TX, USA). All *P* values were two-sided.

## Results

### Study characteristics

**Figure 1:**
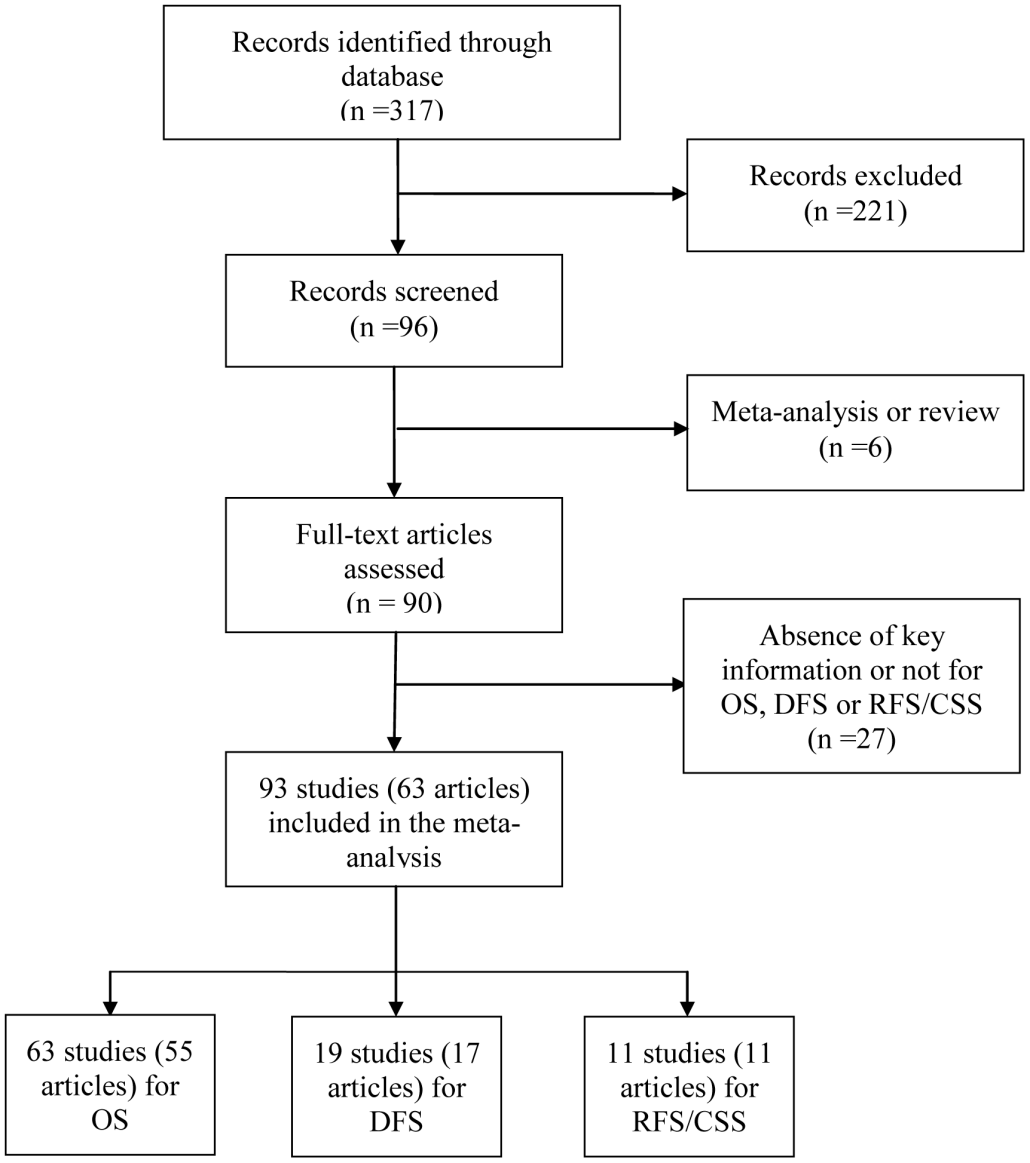
Methodological flow diagram of the review.

After careful read and selection, a total of 63 articles [19], were retrieved according to the inclusion and exclusion criteria. 55 of 63 articles investigated the prognostic role of miR-21 for OS, 17 for DFS, 8 for RFS and 3 for CSS. Schetter et al. [25], Hwang et al. [40] and Akagi et al. [79] presented separate HR by different ethnic background; Mathe et al. [33]_ENREF_34, Liu et al. [51], Toiyama et al. [80], Nielsen et al. [44] and Markou et al. [82] investigated the role of miR-21 in different type of samples; Voortman et al. [43] reported results from two centers. So the data from these studies were considered separately in our analysis. As there were only 3 studies for CSS, we combined the results for CSS and RFS together as RFS/CSS. Thus, a total of 63 studies including 6720 patients evaluating OS, 19 studies including 1965 cases for DFS and 11 studies including 1696 patients for RFS/CSS were considered in this analysis. The detailed screening process was shown in Figure 1.

The main characteristics of eligible studies were listed in Table 1. Ethnicity background of patients were classified as Asian, Caucasian and mixed populations. Cancer types of cases were various, among which lung cancer, pancreatic cancer and gastrointestinal (GI) cancers were mostly investigated. Tissue samples including Frozen or formalin-fixed and paraffin-embedded (FFPE) tissues were used in 53 studies, while 11 studies used circulation samples (plasma, serum or blood) and one study by Ota et al. [47] applied bone marrow samples. Quantitative real-time PCR (qRT-PCR) was widely used in 57 studies and in situ hybridisation (ISH) assay was used in the other 6 studies. The most frequently used cut-off value was the median which was applied in 26 studies and the other values ranged from the mean to the highest quarter value.

**Table 1:** Main characteristics of eligible studies.

Author	Year	Country	Ethnicity	Type	Sample	Stage	Number	Method	Endogenous control	cut-off	Results
**Schetter**	2008	USA/HK	Caucasian/Asian	Colon	Frozen tissue	I-IV	197	qRT-PCR	U6	Highest tertile	OS
**Dillhoff**	2008	USA	Caucasian	Pancreatic	FFPE	NR	80	In Situ Hybridization	U6	Highest score	OS
**Markou**	2008	Greece	Caucasian	NSCLC	Frozen tissue	I-IV	48	qRT-PCR	U6	2-fold	OS and DFS
**Yan**	2008	China	Asian	Breast	FFPE	I-III	113	qRT-PCR	U6	Mean	OS
**Qian**	2009	Italy	Caucasian	Breast	Frozen tissue	I-IV	301	qRT-PCR	U6	NR	OS and DFS
**Busacca**	2010	Italy	Caucasian	Malignant mesothelioma	FFPE	NR	24	qRT-PCR	U6	Median	OS
**Li**	2009	China	Asian	Tongue	Frozen tissue	I-IV	103	qRT-PCR	U6	Median	OS
**Schetter**	2009	HK/USA	Caucasian/Asian	Colon	Frozen tissue	I-IV	196	qRT-PCR	U6	Highest tertile	CSS
**Mathe**	2009	USA,Canada/Japan	Caucasian/Asian	Esophageal	Tissue	I-IV	170	qRT-PCR	U66	Median	OS
**Avissar**	2009	USA	Caucasian	HNSCC	Frozen tissue	I-IV	169	qRT-PCR	U48	Highest quarter	OS
**Zhi**	2010	China	Asian	Astrocytoma	Frozen tissue	I-IV	124	qRT-PCR	miR-16	Median	OS
**Hu**	2011	USA	Caucasian	Esophageal	FFPE	I-IV	158	In situ hybridization	NR	1–3+/0–0.5	OS and DFS
**Gao**	2010	China	Asian	NSCLC	Frozen tissue	I-III	47	qRT-PCR	U6	Median	OS
**Rossi**	2010	USA	Caucasian	CLL	Blood	NR	99	qRT-PCR	U6	Median	OS
**Giovannetti**	2010	Netherlands	Caucasian	Pancreatic	Tissue	I-IV	59	qRT-PCR	U43	Median	OS and DFS
**Hwang**	2010	Korea/Italy	Asian/Caucasian	Pancreatic	Frozen tissue	II-IV	82/45	qRT-PCR	U66/U43	Median	OS,DFS and RFS
**Gao**	2011	China	Asian	SCLC	Frozen tissue	I-III	30	qRT-PCR	U6	Median	OS
**Kulda**	2010	Czech Republic	Caucasian	CRC	Frozen tissue	I-IV	44	qRT-PCR	U6	NR	DFS
**Voortman**	2010	14 countries	Mixed	NSCLC	FFPE	I-III	631	qRT-PCR/In situ hybridization	U66/U6	Median	OS
**Nielsen**	2011	Denmark	Caucasian	Colon/rectum	FFPE	II	129/67	In Situ Hybridization	NR	2-fold	DFS
**Hamano**	2011	Japan	Asian	Esophageal	FFPE	I-IV	98	qRT-PCR	U48	Median	OS
**Radojicic**	2011	Greece	Caucasian	Breast	FFPE	NR	49	qRT-PCR	RNU5A/U6	Median	OS and DFS
**Ota**	2011	Japan	Asian	Breast	Bone marrow	NR	207	qRT-PCR	U6	5.84	OS and DFS
**Walter**	2011	USA	Caucasian	Breast	FFPE	NR	25	qRT-PCR	U6	Median	OS
**Saito**	2011	USA/Norway/Japan	Caucasian/Asian	NSCLC	Frozen tissue	I-II	126/191	qRT-PCR	U66	Median	CSS/RFS
**Shibuya**	2010	Japan	Asian	CRC	Frozen tissue	Dukes:A-D	156	qRT-PCR	U6	Mean	OS and DFS
**Liu**	2012	China	Asian	NSCLC	Frozen tissue	I-IV	70	qRT-PCR	U6	2-fold	OS
**Wang**	2011	China	Asian	NSCLC	Serum	I-III	88	qRT-PCR	U6	5-fold	OS
**Ayerbes**	2011	Spain	Caucasian	Colon or rectum/gastric/pancreas	FFPE	I-IV	32	qRT-PCR	U6	Mean	OS
**Jiang**	2011	China	Asian	Gastric	FFPE	III,IV	55	qRT-PCR	U44	NR	OS
**Nagao**	2012	Japan	Asian	Pancreatic	FFPE	I-IV	65	qRT-PCR	U6	Mean	OS
**Jamieson**	2012	UK	Caucasian	Pancreatic	Frozen tissue	II-III	72	qRT-PCR	U6	Median	OS
**Jiang**	2012	China	Asian	Melanoma	Frozen tissue	I-IV	86	qRT-PCR	U6	Median	OS and DFS
**Liu**	2012	China	Asian	Pancreatic	Serum	I-IV	38	qRT-PCR	NR	NR	OS
**Karakatsanis**	2013	Greece	Caucasian	Hepatocellular	FFPE	I-IV	60	qRT-PCR	U6	Mean	OS
**Gao**	2012	China	Asian	NSCLC	Frozen tissue	I-III	58	qRT-PCR	U6	Median	DFS
**Lee**	2011	Korea	Asian	Breast	FFPE	I-III	109	qRT-PCR	U6	Mean	OS and DFS
**Li**	2012	China	Asian	Prostate	FFPE	II-III	168	in situ hybridization	NR	Score>1	RFS
**Faltejs kova**	2012	Czech Republic	Caucasian	CRC	Frozen tissue	I-IV	44	qRT-PCR	U6	Median	OS
**Faragalla**	2012	Canada	Caucasian	Renal	FFPE	I-III	89	qRT-PCR	U44	NR	OS and DFS
**Zaravinos**	2012	Greece	Caucasian	Bladder	Tissue	NR	77	qRT-PCR	RNU1A1,5A and U6	Median	OS and RFS
**Jung**	2012	USA	Caucasian	Oral	Frozen tissue	NR	17	qRT-PCR	U6	Median	OS
**Le**	2012	China	Asian	Lung	Serum	I-IV	82	qRT-PCR	miR-16	NR	OS
**Xu**	2012	China	Asian	Gastric	Frozen tissue	I-IV	86	qRT-PCR	Let-7a	ROC curve (AUC)	OS
**Osawa**	2011	Japan	Asian	Gastric	FFPE	I-IV	37	qRT-PCR	NR	T/N ratio >1.40	OS
**Papaconstantinou**	2013	Greece	Caucasian	Pancreatic	FFPE	I-IV	88	qRT-PCR	U6	Mean	OS
**Frifeldt**	2012	Denmark	Caucasian	Colon	FFPE	II	520	in situ hybridization	NR	Tertiles	OS and RFS
**Hermansen**	2013	Denmark	Caucasian	Gliomas	FFPE	NR	189	in situ hybridization	NR	NR	OS
**Caponi**	2013	UK/Italy	Caucasian	Pancreatic	FFPE	II-III	81	qRT-PCR	U6	Median	OS and DFS
**Wang**	2013	China	Asian	Pancreatic	Serum	III-IV	177	qRT-PCR	U6	Median	OS
**Komatsu**	2013	Japan	Asian	Gastric	Plasma	I-IV	69	qRT-PCR	NR	Median	CSS
**Amankwah**	2013	USA	Caucasian	Prostate	FFPE	I-IV	65	qRT-PCR	U6	median	RFS
**Chusorn**	2013	Thailand	Asian	Cholangiocarcinoma	Frozen tissue	NR	23	qRT-PCR	U6	Mean	OS
**Huang**	2013	China	Asian	Cholangiocarcinoma	FFPE	NR	41	qRT-PCR	U6	NR	OS and RFS
**Liu**	2013	China	Asian	CRC	Serum	I-IV	166	qRT-PCR	MiR-16	0.0043	OS
**Akagi**	2013	USA,Norway/Japan	Caucasian/Asian	Lung	Frozen tissue	I-II	92/198	qRT-PCR	NR	Median	OS and RFS
**Toiyama**	2013	Japan	Asian	CRC	FFPE/serum	I-IV	166/188	qRT-PCR	miR-16/Cel-miR-39	Youden's index	OS
**Bovell**	2013	USA	Mixed	CRC	FFPE	IV	55	qRT-PCR	U6	NR	OS
**Markou**	2013	Greece	Caucasian	NSCLC	FFPE/plasma	I-IV	40/37	qRT-PCR	miR-191/miR-16	Median	OS and DFS
**Chen**	2013	Taiwan	Asian	CRC	Tissue	I-IV	195	qRT-PCR	U6	Mean	OS
**Ferrajoli**	2013	USA	Caucasian	CLL	Blood	NR	93	qRT-PCR	miR-16	44th percentile	OS
**Menendez**	2013	Spain	Caucasian	CRC	Serum	I-IV	102	qRT-PCR	miR-16	Relative expression>1	OS and DFS
**Kadera**	2013	USA	Caucasian	Pancreatic	Tissue	I-IV	147	qRT-PCR	U6	NR	OS

NSCLC: non-small cell lung cancer; HNSCC: head and neck squamous cell carcinomas; CLL: chronic lymphocytic leukemia; SCLC: squamous cell lung carcinoma; CRC: colorectal carcinoma; ALL: acute lymphoblastic leukemia NR: not reported; FFPE: formalin-fixed and paraffin-embedded; OS: overall survival; DFS: disease-free survival; RFS: recurrence-free survival; CSS: cancer-specific survival.

### Outcomes from eligible studies

**Figure 2:**
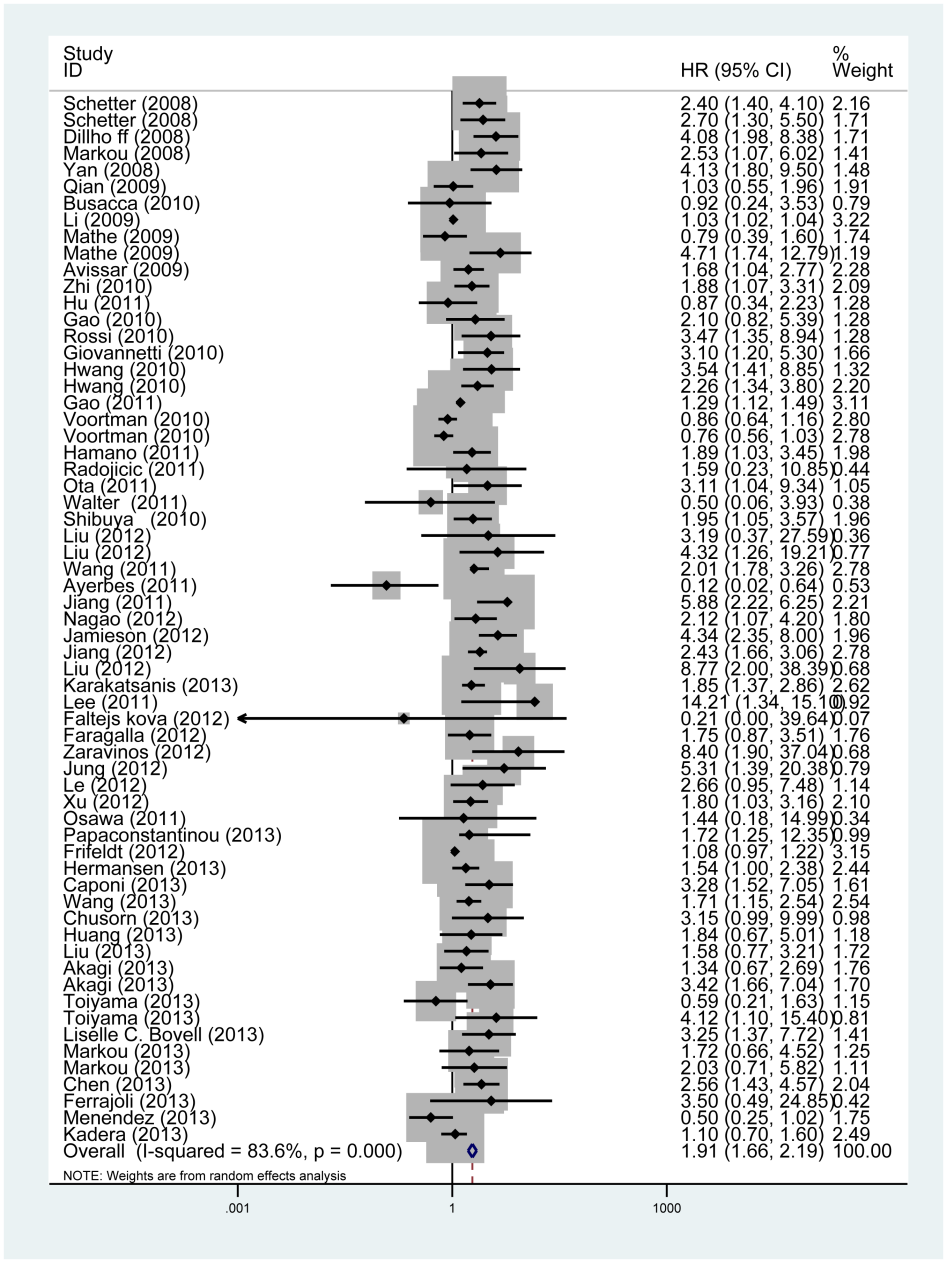
Forrest plots of studies evaluating hazard ratios (HRs) of miR-21 for overall survival.

The main results of this meta-analysis are shown in Table 2. For 63 studies evaluating OS for miR-21, we found high expression of miR-21 predicting a worse outcome with the combined HR of 1.91 (95%CI: 1.66–2.19; P_heterogeneity_<0.001; Figure 2). Similarly predictive roles of miR-21 for DFS and RFS/CSS were also investigated with pooled HR of 1.42 (95% CI: 1.16–1.74; P_heterogeneity_ = 0.001) and 2.2 (95% CI: 1.64–2.96; P_heterogeneity_ = 0.022), respectively.

**Table 2:** Meta-analysis results.

Outcome	Variables	Number of studies	Model	HR (95% CI)	P_heterogeneity_
**OS**	ALL	63	Random	1.91(1.66,2.19)	<0.001
	Cancer type				
	GI	15	Random	1.68(1.12,2.52)	<0.001
	Pancreas	11	Random	2.53(1.82,3.51)	0.003
	Lung	13	Random	1.59(1.2,2.1)	<0.001
	Breast	6	Random	2.55(1.04,6.29)	0.002
	Oral	2	Random	2.02(0.41,9.88)	0.016
	Esophagus	4	Random	1.53(0.74,3.15)	0.018
	Liver	3	Fixed	1.93(1.39,2.69)	0.688
	Ethnicity				
	Asian	29	Random	2.19(1.76,2.73)	<0.001
	Caucasian	29	Random	1.86(1.46,2.37)	<0.001
	Sample				
	Tissue	51	Random	1.87(1.61,2.16)	<0.001
	FFPE	25	Random	1.68(1.29,2.18)	<0.001
	Frozen tissue	23	Random	1.99(1.59,2.49)	<0.001
	Circulation	11	Random	2.06(1.42,2.99)	0.008
	Serum	8	Random	1.94(1.25,3.03)	0.003
	Therapy				
	Adjuvant therapy	7	Random	2.4(1.18,4.9)	<0.001
	Mixed	56	Random	1.85(1.61,2.13)	<0.001
**DFS**	ALL	19	Random	1.42(1.16,1.74)	0.001
	Cancer type				
	GI	5	Random	1.12(0.81,1.55)	0.01
	Pancreas	3	Fixed	2.87(1.89,4.35)	0.524
	Lung	4	Fixed	2.05(1.32,3.18)	0.839
	Breast	4	Fixed	1.1(0.82,1.49)	0.919
	Ethnicity				
	Asian	6	Random	1.62(1.06,2.47)	0.008
	Caucasian	14	Random	1.37(1.07,1.76)	0.006
**RFS/CSS**	ALL	11	Random	2.2(1.64,2.96)	0.022
	Cancer type				
	GI	3	Random	2.5(1.1,5.71)	0.005
	Lung	3	Fixed	2.25(1.57,3.23)	0.605
	Prostate	2	Fixed	2.04(1.17,3.54)	0.957
	Ethnicity				
	Asian	5	Fixed	2.17(1.52,3.09)	0.322
	Caucasian	5	Random	2.1(1.34,3.27)	0.065

OS: overall survival; DFS: disease-free survival; RFS: recurrence-free survival; CSS: cancer-specific survival; GI: gastrointestinal; FFPE: formalin-fixed and paraffin-embedded.

Subgroup analyses by cancer type showed that elevated miR-21 yielded a worse OS in GI cancers (HR = 1.68, 95%CI: 1.12–2.52; P_heterogeneity_<0.001), lung cancer (HR = 1.59, 95%CI: 1.2–2.1; P_heterogeneity_<0.001), breast cancer (HR = 2.55, 95%CI: 1.04–6.29; P_heterogeneity_ = 0.002), pancreatic cancer (HR = 2.53, 95%CI: 1.82–3.51; P_heterogeneity_ = 0.003) and liver cancer (HR = 1.93, 95%CI: 1.39–2.69; P_heterogeneity_ = 0.688); a worse DFS in lung cancer (HR = 2.05, 95%CI: 1.32–3.18; P_heterogeneity_ = 0.839) and pancreatic cancer (HR = 2.87, 95%CI: 1.89–4.35; P_heterogeneity_ = 0.524); a poorer RFS/CSS in GI cancers (HR = 2.5, 95%CI: 1.1–5.71; P_heterogeneity_ = 0.005), lung cancer (HR = 2.25, 95%CI: 1.57–3.23; P_heterogeneity_ = 0.605) and prostate cancer (HR = 2.04, 95%CI: 1.17–3.54; P_heterogeneity_ = 0.957).

In the subgroup analyses by ethnicity, we found that no matter the cases were Asian or Caucasian, the high expression of miR-21 was still a significantly poor predictor for OS (Asian: HR = 2.19, 95%CI: 1.76–2.73; P_heterogeneity_<0.001; Caucasian: HR = 1.86, 95%CI: 1.46–2.37; P_heterogeneity_<0.001), DFS (Asian: HR = 1.62, 95%CI: 1.06–2.47; P_heterogeneity_ = 0.008; Caucasian: HR = 1.37, 95%CI: 1.07–1.76; P_heterogeneity_ = 0.006) and RFS/CSS (Asian: HR = 2.17, 95%CI: 1.52–3.09; P_heterogeneity_ = 0.322; Caucasian: HR = 2.1, 95%CI: 1.34–3.27; P_heterogeneity_ = 0.065).

Further analyses of studies evaluating OS by sample type also revealed that high expression of miR-21 remained to be a worse prognostic marker regardless of sample source (tissue sample: HR = 1.87, 95%CI: 1.61–2.16; P_heterogeneity_<0.001; circulation sample: HR = 2.06, 95%CI: 1.42–2.99; P_heterogeneity_ = 0.008). In addition, high miR-21 in FFPE (HR = 1.68, 95%CI: 1.29–2.18; P_heterogeneity_<0.001) and frozen tissue (HR = 1.99, 95%CI: 1.59–2.49; P_heterogeneity_<0.001) showed consistent results. Pooled results of 8 studies that explored serum miR-21 also revealed negative prognostic role of increased miR-21 (HR = 1.94, 95%CI: 1.25–3.03; P_heterogeneity_ = 0.003)

A total of seven studies [27], [39], [40], [43], [53], [82] investigated the prognostic role of miR-21 in the patients who received adjuvant therapy which yielded a significantly pooled HR of 2.4 (95%CI: 1.18–4.9; P_heterogeneity_<0.001).

### Publication bias

**Figure 3:**
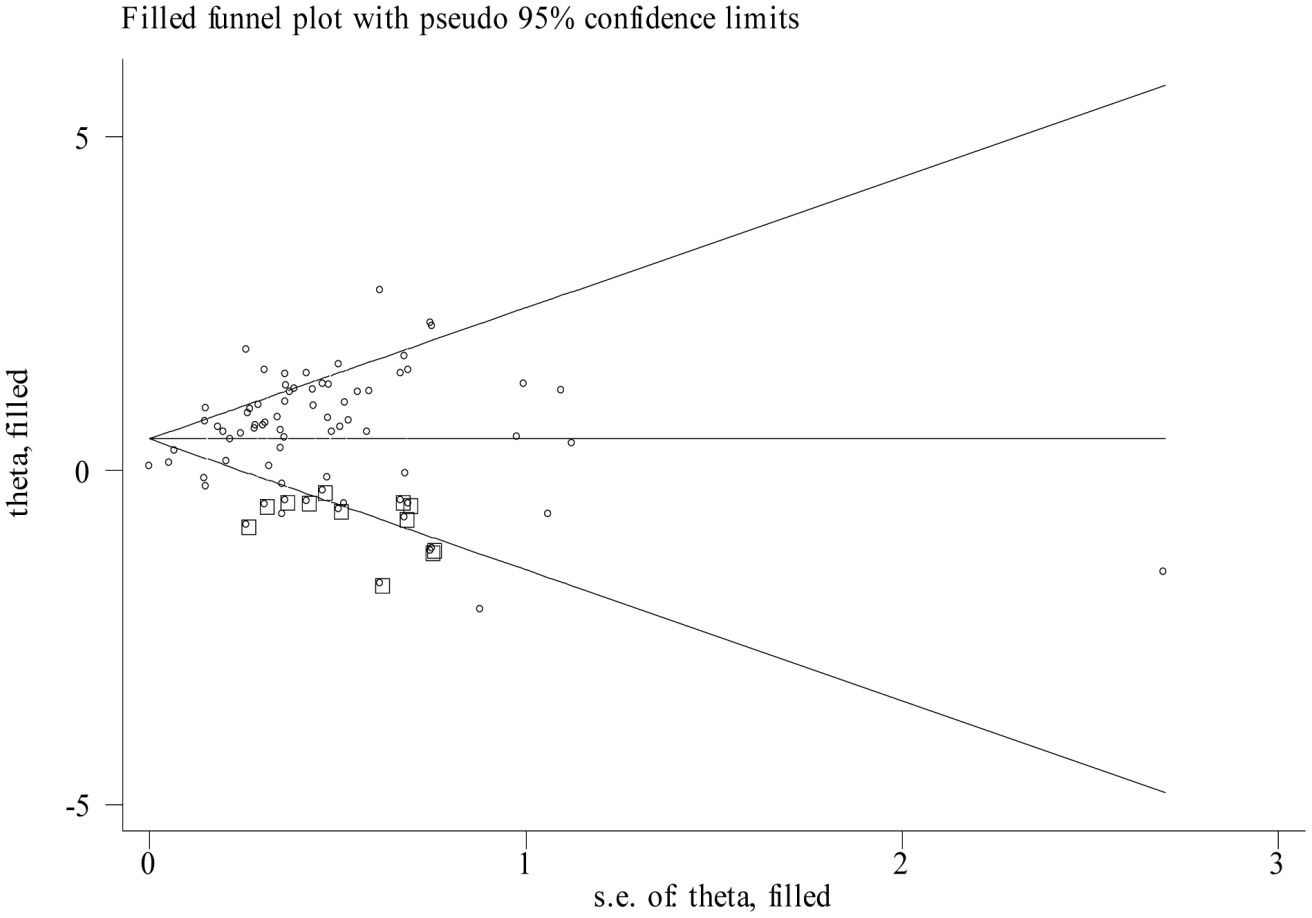
Funnel plot adjusted with trim and fill method for overall survival. Circles: included studies. Diamonds: presumed missing studies.

Begg's funnel plot and the Egger's linear regression test were used to assess publication bias. However, the funnel plots were asymmetric and the P values of Egger's test for OS, DFS and RFS/CSS were <0.001, 0.011 and 0.003, respectively. Thus, a trim and fill method was conducted and pooled HRs were recalculated with hypothetically non-published studies to evaluate the asymmetry in the funnel plots. The recalculated HRs did not change significantly for OS (HR = 1.61, 95%CI: 1.41–1.83; P_heterogeneity_<0.001;Figure 3) and RFS/CSS (HR = 2.01, 95%CI: 1.54–2.77; P_heterogeneity_ = 0.018). But the prognostic role of high expression of miR-21 for DFS was weaken with a recalculated HR of 1.11 (95%CI: 0.9–1.38; P_heterogeneity_<0.001).

## Discussion

MiR-21, a well-known onco-miR, is up-regulated in most malignancies. Acting on various target genes such as PTEN [87] and PDCD4 [18], miR-21 plays an important role in the process of cell proliferation, migration, invasion, drug resistance [88] and so on. It has been reported that miR-21 could regulate Ras/MEK/ERK pathway so to influence the tumor formation. Moreover the incidence of lung tumors is higher in miR-21 overexpression mice, while lower in miR-21 knockout mice [89]. Additionally, miR-21 has been proposed as a marker of cancers for diagnosis in circulation [90], [91], stool [92] and sputum [93], prediction in therapy response [59] and prognosis of patients.

Nair et al. [94] systematically reviewed and synthesized that miRNAs showed promising associations with outcomes of various cancers. As the first meta-analysis [95] of miR-21 related to outcomes of various cancers, Fu et al. retrieved 17 studies and found higher level of miR-21 might be associated with poorer clinical outcome, especially in subgroup of head and neck squamous cell carcinoma and digestive carcinoma. Recently, Wang et al. [96] analyzed the value of circulating miR-21 and yielded a conclusion that circulating miR-21 might act as a significantly prognostic biomarker but not be suitable for a sensitive diagnostic biomarker. However, the number of studies included in these analyses was relatively small and the obtained results might not be powerful. In terms of this, we performed this updated meta-analysis including 63 articles and demonstrated that high expression of miR-21 was a significant marker for predicting worse outcomes of various cancers (HR was 1.91, 2.2 and 1.42 for OS, RFS/CSS and DFS, respectively). Subgroup analyses revealed that high expression of miR-21 could predict a worse OS in GI tumors, pancreatic cancer, lung cancer, breast cancer and liver cancer, a worse DFS in pancreatic cancer and lung cancer and poor RFS/CSS in GI tumors, lung cancer and prostate cancer. Regardless of the ethnicity background or sample source, high expression level of miR-21 was a significantly negative prognostic marker for various malignancies. As publication bias was observed, a trim and fill method was adopted to calculate the adjusted HRs. The results for OS and RFS/CSS did not change, but the results for DFS were altered.

Recently, many studies demonstrated that miRNAs including miR-21 had great potential as biomarkers for various cancers. However, several problems should be well solved before utilizing them as diagnostic or prognostic biomarkers in the clinical. As is known, non-invasive circulation sample (plasma/serum) or body fluid sample could be obtained more conveniently than tissue sample. However, studies using different types of samples may yield different results [51]. Tsujiura et al. [91] found that some individuals might even have opposite tendency of the expression levels of miRNAs in tumor tissue and plasma. Now, many studies have investigated the clinical impact of miRNAs from exosomes which were small membrane vesicles containing proteins and nucleotides [97]. In our study, it is pleasing that high expression of miR-21 in the tissue (FFPE/frozen tissue) or circulation both predicted poor outcomes. Thus, we might assume that patients with high expression of miR-21 from any type of sample might suffer worse clinical outcomes. Yet, normalization among different studies was not consistent. The internal controls used for tissue samples are relatively consistent ranging from U6 to U44, while there is no consensus on suitable small RNA reference genes for circulation or body fluid sample. MiR-16 was used as a reference gene in some studies [66], [78]. But the optimal way for miRNA normalization in circulation or body fluid sample is probably the spiked-in normalization method [98]. Therefore, future studies focusing on the consistent normalization are warranted. In addition, as biomarkers, a panel of miRNAs might be more sensitive and specific than a single miRNA [99], [100]. The combination of miR-21 and some specific miRNAs might elevate its predictive power. Finally, methods for detecting miRNAs were diverse, among which RT-PCR was one of the most widely used approaches. Nevertheless many new methodologies emerged, such as the next-generation sequencing approach [101] and the electrochemical approach [102]. In short, a proper method for clinical application should be less expensive, reproducible, stable and with high sensitivity and specificity. Accordingly, great efforts should be made in the future to apply miRNAs including miR-21 as reliable biomarkers in the clinical.

Several limitations of this study should be considered. First, the studies retrieved in our study were limited in English, which might partially contribute to the observed publication bias. By conducting the trim and fill method, we found that the pooled results did not change significantly except for DFS. Thus, attention should be paid to the prognostic role of miR-21 for DFS. Second, different countries, cancer types, methods and other variables might contribute to the relatively large heterogeneity in this study. Third, the number of studies investigating some special types of cancer was small. For instance, there was only one study focusing on mesothelioma [30]. More studies on these cancers are needed in the future.

In conclusion, the evidence from the meta-analysis revealed that high expression level of miR-21 was a negative predictor for survival in various cancers, especially for OS and RFS/CSS. However, our results should be considered with caution due to the limitations listed above. To better understand and use miRNAs as biomarkers in the clinical, more large-scale and standard investigations are worth conducting.

## Supporting Information

Checklist S1 **PRISMA Checklist.**
(DOC)
